# Generation of Self‐Organizing Macrovascular Constructs by Bioprinting Human iPSC‐Derived Mesodermal Progenitor Cells

**DOI:** 10.1002/advs.76018

**Published:** 2026-06-09

**Authors:** Leyla E. Dogan, Nathaly A. Chicaiza‐Cabezas, Florian Kleefeldt, Philipp Wörsdörfer, Jürgen Groll, Süleyman Ergün

**Affiliations:** ^1^ Institute of Anatomy and Cell Biology Julius‐Maximilians‐University of Würzburg Würzburg Germany; ^2^ Department of Functional Materials For Medicine and Dentistry at the Institute for Functional Materials and Biofabrication (IFB) and Bavarian Polymer Institute (BPI) Julius‐Maximilians‐University of Würzburg Würzburg Germany; ^3^ Atlas University Research Center (ARC) Atlas University İstanbul Turkey

**Keywords:** bioprinting, induced pluripotent stem cells, macrovessels, microvessels, organoids

## Abstract

Vascularization remains a major obstacle in tissue engineering. Here, we introduce a bioprinting strategy to generate centimeter‐scale, self‐organizing “mother vessel” constructs from iPSC‐derived hiMPCs. By optimizing bioink composition, printing was accomplished in a single‐step approach. Within one week, hiMPCs differentiated into both CD31^+^ endothelial and αSMA^+^ mural cells, driving the morphogenesis of the vessel wall comprising intima, media and adventitia. The remaining hiMPCs formed a mesodermal tissue around the vessel wall. Remarkably, CD34^+^, CD150^+^, and IBA1^+^ progenitors were detected within the mother vessel construct. The vessel wall reached a median thickness of approximately 150 µm within the first 10 days and exhibited collagen III and elastic fibers. Furthermore, eNOS expression was detected in endothelial cells. Co‐culture of the “mother vessel” with prevascularized organoids resulted in interconnection between organoid‐derived microvessels and those of the mother vessel. Angiogenic sprouts extended into the surrounding mesodermal tissue. Monocyte adhesion to luminal endothelial cells was significantly increased following TNFα stimulation. After integration into a bioreactor, the mother vessel withstood applied flow without detectable leakage. In summary, we established a developmentally inspired platform that bridges macro‐ and microvascularization. This approach may pave the way toward perfusable, vascularized large‐scale tissue constructs, addressing a major bottleneck in regenerative biofabrication.

## Graphical abstract

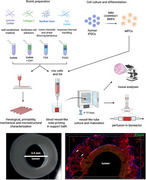

## 1 Introduction

A functional and well‐organized vascular network is essential for maintaining tissue homeostasis, ensuring a continuous supply of oxygen and nutrients while removing carbon dioxide and other metabolic by‐products. The vascular system forms a branching hierarchical network that ranges from large arteries and veins to smaller arterioles, capillaries, and venules. Except for capillaries, all blood vessels share a characteristic triple‐layered wall: the inner intima is formed by endothelial cells (ECs) and their basement membrane lining the vessel lumen, the media contains smooth muscle cells (SMCs) that mediate contractile functions, and the outer adventitia harbors fibroblasts as well as stem and progenitor cells [1, 2].

Vascularization is essential for advancing 3D in vitro tissue models that are used to study human development, physiology, and disease, and may serve as tissue replacement in the future. However, achieving proper vascularization remains one of the major challenges in bioengineering across tissues. Current approaches in vascular tissue engineering mostly combine adult vascular cell types such as ECs, SMCs, and fibroblasts with supportive scaffold materials. These scaffolds range from decellularized native vessels [3, 4] to synthetic and natural hydrogels (e.g., alginate, gelatin methacryloyl (GelMA), poly(ethylene glycol) diacrylate (PEGDA), collagen, and fibrin) [5, 6, 7, 8, 9, 10]. The matrices are intended to provide a supportive environment for cell adhesion, survival, and extracellular matrix formation that enables the fabrication of tubular constructs. However, most of the existing bioprinted vascular models published so far neither reproduce the multilayered vascular wall of native vessels nor a hierarchical network organization as observed in vivo which largely limits their translational relevance.

During embryonic development, de novo vessel formation, termed vasculogenesis, is driven by endothelial cells that differentiate from mesodermal progenitors and subsequently self‐organize into an immature capillary plexus at intra‐ and extraembryonic sites [11]. These processes are mainly governed by vascular endothelial growth factor A (VEGF‐A). Likewise, the embryonic dorsal aorta, initially formed as paired vessels that subsequently fuse into a single dorsal aorta, develops through similar mechanisms and is initially composed solely of an endothelial tube. The entire vascular network then undergoes extensive remodeling, ultimately giving rise to a unified vascular system consisting of interconnected macro‐ and micro‐vessels organized in a hierarchical manner.

Reproducing these morphogenetic processes in vitro by integrating bioprinting with the intrinsic self‐organizing capacity of hiMPCs therefore represents a promising biologically inspired strategy to achieve structurally and functionally mature macrovessels in centimeter‐ and millimeter‐scale that are hierarchically interconnected with engineered microvascular networks in tissues, e.g., organoids.

In line with this, we previously succeeded in generating mesodermal progenitors (hiMPCs) from human iPSCs that exhibited the capacity to differentiate in vitro into all cell types required for blood vessel formation, thereby eliminating the need for pre‐differentiated vascular cells. Moreover, following extrusion‐based bioprinting, these hiMPCs retained their multipotency, underwent spontaneous differentiation, and self‐organized into hierarchically structured, blood vessel‐like constructs that recapitulate key features of early embryonic vascular development [5].

However, their intrinsic self‐organizing capacity alone is not sufficient to form larger conduit vessels (>1 mm inner diameter) that are required for functional perfusion or surgical integration with the host vasculature in vivo. To overcome this limitation, we employed 3D bioprinting to predefine millimeter‐scale “mother vessels” designed to interconnect with the vascular networks of prevascularized surrounding tissues, such as organoids, and to serve as defined in‐ and outflow channels for these tissue constructs. To implement this biologically driven concept, we evaluated different hydrogel formulations for their suitability in extrusion bioprinting and vascular morphogenesis. We were able to identify a blend matrix that provided a balance between printability, mechanical stability, and cellular compatibility. Using this optimized formulation, we successfully printed centimeter‐scale “mother vessel” constructs that exhibited early vascular wall layering and were suitable for initial perfusion experiments. Furthermore, direct co‐culture with prevascularized organoids demonstrated early structural interconnection with the “mother vessel” representing a first step toward assembling larger tissues through a modular building‐block approach. This single‐step, developmentally guided strategy offers a simple approach to generate macro‐scale vascular tissues that recapitulate key aspects of embryonic vasculogenesis that will facilitate to bridge the gap between large host vessels and microvascular networks within engineered tissues. Moreover, a thick matrix layer containing hiMPCs at various stages of differentiation, which display immunophenotypic markers characteristic of mesodermal, endothelial, and hematopoietic lineages, surrounds the “mother vessel”. It is conceivable that mesodermal tissue containing different progenitor cell types serves as a reservoir that delivers vascular wall cells required for vascular remodeling and morphogenesis, as well as for further tissue vascularization in response to mechanical and physiological demands, such as those for vessels with varying wall thickness and matrix composition (e.g., large and medium‐sized arteries and veins).

## 2 Material and Methods

### 2.1 Cell Culture and Differentiation

Human induced pluripotent stem cells (hiPSCs) were generated from commercially available human dermal fibroblasts using the hSTEMCCA lentiviral reprogramming vector as described previously [12, 13]. hiPSCs were maintained on Matrigel‐coated culture dishes (hESC‐qualified matrix, 354277, Corning, New York, USA) in StemMACS iPS Brew medium (130‐107‐086, Miltenyi Biotec, Bergisch Gladbach, Germany) with daily medium exchange. For routine passaging, cultures at approximately 80% confluence were dissociated into single cells using StemPro Accutase (A6964, Merck, Temecula, USA) for 5 min at 37°C. The cells were reseeded onto Matrigel‐coated dishes in StemMACS medium supplemented with 10 nm thiazovivin (TZ, T9753, LC Labs, Woburn, MA, USA) to enhance cell survival and attachment.

HiPSCs were differentiated into mesodermal progenitor cells (hiMPCs) using a previously established differentiation protocol [14]. Confluent hiPSC cultures were dissociated with Accutase, counted and seeded on Matrigel‐coated six‐well plates (3.5 × 10^5^ cells/cm^2^). Cells were maintained for 24 h in StemMACS iPS‐Brew medium supplemented with 10 nm TZ. The medium was then replaced with mesodermal induction medium (Advanced DMEM/F‐12, 126–34010,

Thermo Fisher Scientific) containing 0.2 mm L‐glutamine (G7313, Merck), 60 µg/mL ascorbic acid (A4544, Sigma–Aldrich, St. Louis, USA), 10 µm CHIR99021 (SML1046, Merck), 25 ng/mL BMP4 (PHC9534, Thermo Fisher Scientific) for three days at 37°C and 5% CO_2_ with daily medium replacement. After induction, cells were dissociated again with Accutase and collected for bioprinting. Printed “mother vessel” constructs were transferred to culture flasks and maintained in vascular growth medium (Advanced DMEM/F‐12 supplemented with 5% heat‐inactivated FCS (S00H81000C, Biowest, Karlruhe, Germany), 1% penicillin/streptomycin (P0781, Merck), 10 nm TZ, 0.2 mm L‐glutamine, 60 µg/mL ascorbic acid and 60 ng/mL VEGF‐A (10020, Thermo Fisher Scientific)) at 37°C and 5% CO_2_.

3D prevascularized mesodermal organoids were generated as described in a previously published protocol [14]. However, instead of using 96‐well plates, custom‐made agarose molds were utilized. These honeycomb‐shaped microwell molds were generated using a 2% (w/v) agarose solution (840004, Biozym, Hessisch Oldendorf, Germany) prepared in filtered Ampuwa (1080181, Freseneus Kabi, Bad Homburg, Germany). The hot agarose solution was poured over 3D‐printed negative molds placed in wells of a 12‐well plate and allowed to solidify for 1 h at RT. Subsequently, the agarose was gently separated from the 3D‐printed negatives, resulting in molds with 159 microwells, each approximately 700 µm in diameter. The agarose molds were filled with DMEM supplemented with 1% penicillin/streptomycin and sterilized under UV light O/N. Prior to seeding, DMEM was removed and replaced by MACS TZ medium supplemented with TZ (1:4000). 4 × 10^4^ cells of a hiPSCs single‐cell suspension were seeded into each mold. Cells were cultured for 1 day in MACS TZ medium, followed by 3 days in mesoderm induction medium (MIM) as described above. On day 4, the medium was replaced with vascular growth medium, in which the organoids were maintained until they reached the size (approximately 300 µm) at culture day 7.

### 2.2 Bioink Preparation

#### 2.2.1 Material Synthesis

Porcine and piscine gelatin methacryloyl (GelMA) were synthesized following a protocol published previously [15]. Briefly, gelatin from porcine skin or cold‐water fish skin (G7041 and G1890, Sigma–Aldrich) was dissolved in phosphate‐buffered saline (1x PBS, D1408, Sigma–Aldrich) at a concentration of 10% w/v at 37°C. Methacrylic anhydride (276685, Sigma–Aldrich) was added dropwise (0.6 g per g of gelatin) under continuous stirring, and the reaction was allowed to proceed for 1 h at 37°C. Unreacted methacrylic anhydride was removed via centrifugation, and the supernatant was diluted twofold with prewarmed Milli‐Q water (37°C). The solution was transferred to a dialysis membrane (MWCO 3.5 kDa) and dialyzed against Milli‐Q water for three days. The resulting GelMA solution was then lyophilized and stored at −20°C until further use.

### 2.3 GelMA Preparation

Porcine GelMA (5% w/w) was dissolved in PBS under magnetic stirring (200 rpm) at 47°C. After complete dissolution, lithium phenyl‐2,4,6‐trimethylbenzoylphosphinate (LAP, 0.1% w/v; 900889, Sigma–Aldrich) was added, and the solution was stirred for an additional 10 min. The resulting GelMA‐LAP solution was maintained in a 37°C water bath until further use. For bioink preparation, hiMPCs were detached, counted, and resuspended in the prewarmed GelMA solution at a final concentration of 2 × 10^7^cells/mL.

### 2.4 GelMA+Col I Preparation

Collagen type I (Col I, 4.7 mg/mL; rat tail, 08–115, Merck) was neutralized according to the manufacturer's instructions. The neutralized Col I was blended with 5% (w/w) porcine GelMA at a 1:4 ratio using a positive displacement pipette, resulting in a final composition of 3.75% (w/w) GelMA and 0.68 mg/mL Col I. For bioink preparation, hiMPCs were detached, counted, and resuspended in the neutralized collagen solution. The cell‐collagen suspension was then combined with 5% porcine GelMA in the same 1:4 ratio to yield the final bioink containing 2 × 10^7^ cells/mL.

### 2.5 FGX Preparation

Piscine GelMA (3% w/w) and porcine GelMA (2% w/w) were dissolved in vascular growth medium under magnetic stirring (200 rpm) at 47°C. After complete dissolution, LAP (0.1% w/v) and xanthan gum (0.5% w/v) (06‐003, Cosphaderm X34, Cosphatec, Hamburg, Germany) were added. The solution was stirred for 45 min (100 rpm, 47°C) to ensure homogeneity. The resulting blend (FGX) was maintained in a 37°C water bath until cell addition. For bioink preparation, hiMPCs were detached, counted, and resuspended in the prewarmed FGX blend at a final concentration of 2 × 10^7^ cells/mL.

### 2.6 FGXC Preparation

Piscine GelMA (6% w/w) and porcine GelMA (4% w/w) were dissolved in vascular growth medium under magnetic stirring (200 rpm) at 47°C. After complete dissolution, LAP (0.2% w/v) and xanthan gum (1% w/v) were added, and the mixture was stirred for 45 min at 100 rpm and 47°C to ensure homogeneity. The resulting blend (2× FGX) was subsequently mixed at a 1:1 ratio with 1% Fibercoll‐Flex N bioink (500069016, Viscofan Bioengineering, Weinheim, Germany), prepared according to the manufacturer's instructions. The final composite blend (FGXC) contained 3% piscine GelMA, 2% porcine GelMA, 0.5% xanthan gum, 0.1% LAP, and 0.5% fibrillar Col I. For bioink preparation, hiMPCs were detached, counted, and resuspended in the prewarmed FGXC blend at a final concentration of 2 × 10^7^ cells/mL.

### 2.7 Support Bath Preparation

Hyaluronic acid sodium salt (0.2% w/v, 80–100 kDa, FH63427, Biosynth, Staad, Switzerland) was dissolved in HEPES buffer (H4034, Sigma–Aldrich, pH 7.4, 10 mm) under magnetic stirring (500 rpm, RT). After complete dissolution, the solution was passed through a 0.2 µm filter. UV‐sterilized xanthan gum (1% w/v) was added, and the mixture was stirred (700 rpm, RT) until a homogeneous solution was obtained.

### 2.8 Rheological Characterization

Rheological measurements were performed using an MCR 702 MultiDrive rotational rheometer (Anton Paar, Graz, Austria) equipped with a 25 mm parallel‐plate geometry at 25°C. For bioink characterization, shear rate sweep tests were carried out to determine the apparent viscosity by increasing the shear rate from 0.001 to 100/s over 300s. Temperature‐dependent viscoelastic behavior was analyzed by temperature sweep measurements of complex viscosity (η^*^), storage modulus (G′), and loss modulus (G″). The temperature varied at a rate of 0.01°C/s in four steps: (1) heating from 15°C to 45°C, (2) isothermal hold at 45°C, (3) cooling from 45 to 15°C, and (4) isothermal hold at 15°C. For the embedding medium, amplitude sweep measurements were conducted over a strain range of 0.01%–1000% at a constant angular frequency of 10 rad/s. Thixotropic behavior and stress recovery were evaluated in a five‐step test sequence: (1) low shear stress for 60s, (2) high shear stress for 10s, (3) low shear stress for 60s, (4) high shear stress for 10s, and (5) low shear stress for 60s. Data visualization performed using Prism11 Software (GraphPad, LaJolla, CA, USA).

### 2.9 Embedded 3D Bioprinting

Tubular constructs were designed in Autodesk Fusion 360 (Autodesk, San Francisco, CA, USA) as cylinders with a diameter of 4.6 mm and heights of 5, 10, or 20 mm. The resulting CAD models were exported and sliced in Slic3r using a 0% infill and a layer height of 0.2 mm. Cell‐laden bioinks were transferred into 3 mL syringe barrels (Nordson), sealed with tip caps and centrifuged at 1200 rpm for 2 min at RT to remove air bubbles. Subsequently, the tip caps were replaced with 22G (1 inch, 0.41 mm inner diameter) or 20G (1 inch, 0.61 mm inner diameter) needles, and the prepared syringes were mounted into the printhead of a BIO X bioprinter (CELLINK, Gothenburg, Sweden). The specific printing parameters used for each bioink formulation are summarized in Table 1.

**TABLE 1:** Printing Parameters for different bioink formulations.

Bioink	Pressure [kPa]	Speed [mm/min]	Needle Gauge	Temperature [°C]
GelMA	15–25	300	22 G	18–24
GelMA+Col I	15–20	300	22 G	18–24
FGX	4–5	300	22 G	RT
FGXC	6–7	360	20 G	RT

For 5 and 10 mm tubular constructs, 12‐well plates were filled with the support bath, and one tube was printed per well. The entire plate was then exposed to 405 nm light (54 mW cm^−^^2^) for 3 min to induce photopolymerization. After crosslinking, the printed tubes were carefully removed from the support bath, rinsed in PBS to remove residual xanthan gum, and transferred into cell culture medium. For 20 mm constructs, glass vials were filled with the support bath, and the longer tubes were printed directly inside the vials. Crosslinking and washing were performed as described above.

### 2.10 Printability Characterization

The inks were prepared and kept at RT for at least 20 min before printing. The printability tests were performed using a 22G needle nozzle, 0.25 inch. A filament fusion test (FFT) was used to define the category of the ink, according to Lamberger et al. [16], and a semi‐quantitative evaluation based on the pore geometry (Pr) was used to characterize printed grids, according to Ouyang et al. [17]. Data visualization performed using Prism11 Software.

### 2.11 Mechanical Characterization

Compression testing was performed using a dynamic mechanical tester (ElectroForce 5500, Bose; TA Instruments, New Castle, DE, USA). Hydrogel samples were extruded into molds, crosslinked under 405 nm light (3 min, 54 mW cm^−2^) and tested using a 250 g load cell. The Young's modulus was determined from the slope of the linear region of the stress–strain curve within the viscoelastic range. Data visualization and statistical analysis were performed using Prism11 Software.

### 2.12 Cryo‐Scanning Electron Microscopy

The microstructure of the hydrogels was examined using cryo‐scanning electron microscopy (cryo‐SEM). Samples were extruded, crosslinked, and analyzed immediately after preparation. In addition, FGXC samples were incubated for 2 and 6 days prior to analysis to assess structural alterations over time. For cryo‐SEM preparation, hydrogel specimens were placed between aluminum plates (3 mm diameter) and rapidly frozen in slushed liquid nitrogen at −210°C. The frozen samples were transferred using an EM VCT100 cryo‐shuttle (Leica Microsystems) into an ACE 400 sputter coater (Leica Microsystems) at −140°C. After removing one aluminum plate, the exposed fracture surface was etched for 15 min at −85°C under vacuum (<1×10^−^^3^ mbar). The surface was then sputter‐coated with 3 nm platinum and transferred to a Crossbeam 340 SEM (Zeiss). Imaging was performed at −155°C with an accelerating voltage of 8 kV.

### 2.13 Cell Viability

To assess cell viability during culture, live/dead staining was performed using Calcein AM (2 µm; C3099, Life Technologies) and Ethidium Homodimer‐1 (EthD‐1, 4 µm; E1169, Life Technologies) according to the manufacturer's instructions. Printed tubular constructs from independent experiments were analyzed at days 1, 3, and 7. At each time point, three constructs were rinsed three times 5 min with PBS on a rocker to remove residual medium. Samples were then fully immersed in freshly prepared staining solution and incubated at 37°C for 1 h. Following incubation, the staining solution was replaced with PBS, and the constructs were washed three times for 15 min each on a rocker. Fluorescence imaging was acquired using a confocal laser scanning microscope (Nikon Eclipse Ti with Nikon NIS‐Elements software version 4.13.05, Nikon, Tokyo, Japan or a Stellaris 8 confocal microscope (Leica Microsystems, Wetzlar, Germany). Images were analyzed using Fiji software to quantify live and dead cell fractions (https://doi.org/10.1038/nmeth.2019). Data visualization and statistical analysis (Student T‐test) were performed using Prism5 Softcutware (GraphPad, LaJolla, CA, USA).

### 2.14 Immunofluorescence

To evaluate the cellular phenotype prior to bioprinting, cells maintained in 2D culture were fixed with 4% paraformaldehyde (PFA; P6148, AppliChem, Darmstadt, Germany) for 15 min at RT. After fixation, samples were blocked for 1 h at RT in 4% bovine serum albumin (BSA; A1391, AppliChem) blocking buffer solution. For intracellular markers, 0.1% Triton X‐100 (T8787, Sigma–Aldrich) was added to the blocking buffer to permeabilize cell membranes, whereas surface markers were stained without detergent. Cells were then incubated O/N at 4°C with primary antibodies diluted in blocking buffer. The following antibodies were used: OCT4 (sc‐5279, Santa Cruz Biotech, Dallas, TX, USA), SOX2 (MAB2018, Santa Cruz Biotech), Brachyury/T (AF2085, Santa Cruz Biotech), and VEGFR‐2 (sc‐48161, Santa Cruz Biotech). After washing three times with PBS, cells were incubated for 1 h at RT with Cy2‐ or Cy3‐conjugated secondary antibodies (Dianova, Hamburg, Germany) diluted in PBS. Fluorescence imaging was performed using a Biorevo fluorescence microscope (Keyence, Osaka, Japan).

For histological analysis, bioprinted tubular constructs were washed three times in PBS (10 min each) and fixed O/N in freshly prepared 4% PFA at 4°C. Fixed samples were washed in PBS (3 × 30 min) to remove residual fixative and stored in 70% ethanol (S642, Nordbrand, Darmstadt, Germany) until paraffin (1.116092504, CarlRoth, Darmstadt, Germany) embedding. Sections (10 µm) were deparaffinized, rehydrated and stained with hematoxylin (50837, Chroma, Darmstadt, Germany) and eosin (A0822, Chroma, Darmstadt, Germany) (H&E) or processed to visualize collagenous matrix components. For immunofluorescence on paraffin sections, antigen retrieval was performed in 10 mm sodium citrate buffer (pH 6.0). Sections were blocked for 1 h at RT in 4% BSA. For intracellular markers, 0.1% Triton X‐100 was added. After blocking, sections were incubated overnight at 4°C with primary antibodies diluted in blocking buffer. The following primary antibodies were used: CD31 (M0823, DAKO, Agilent, Santa Clara, CA, USA), αSMA (ab5694, Abcam, Cambridgeshire, UK), CD34 (130‐105‐830, Miltenyi Biotech), CD150 (PA5‐21123, Invitrogen), CD44 (103002, BioLegend, San Diego, CA, USA), Collagen III (SAB4200749, Merck, USA), Collagen IV (ab6586, Abcam), CD45 (13‐9457‐82, Invitrogen), IBA1 (019‐19741, Wako/Fuji Film, Neuss, Germany) and eNOS (R&D Systems, AF970, Germany).

After washing three times in PBS, sections were incubated for 1 h at RT with fluorophore‐conjugated secondary antibodies (Dianova, Hamburg, Germany): goat anti‐rabbit Cy2 (111‐225‐144), goat anti‐rabbit Cy3 (111‐165‐003), goat anti‐mouse Cy2 (115‐225‐146), goat anti‐mouse Cy3 (115‐165‐003) and goat anti‐rat Cy5 (112‐175‐143). Nuclei were counterstained with DAPI (2360276, Merck). Stained sections were imaged using a Nikon Eclipse Ti confocal laser scanning microscope (Nikon).

For vibratome sections, printed tubular constructs were washed three times with PBS (10 min each) on a shaker and subsequently fixed in 4% PFA (in 0.1 m PBS O/N at 4°C). Excess fixative was removed by washing the samples three times with PBS for 30 min each. The fixed constructs were embedded in warm 1% agarose prepared in distilled water (dH_2_O). After solidification, the agarose blocks were mounted onto the cutting platform using superglue and sectioned (300 µm) with a vibratome (VT1000S, Leica Microsystems, Germany). Blocking was performed for 2 h at RT, followed by overnight incubation with primary antibodies at 4°C and 3 h incubation with Cy2‐ or Cy3‐conjugated secondary antibodies at RT in PBS.

### 2.15 H&E Staining

Following deparaffinization slides were immersed in hematoxylin solution (Chroma, 50837, Waldeck GmbH, Münster, Germany) for 10 min, rinsed briefly with distilled water, and then washed for 10 min under running tap water. After a final rinse with distilled water, cytoplasmic counterstaining was performed with eosin for 10 min. Subsequently, slides were dehydrated through graded ethanol (96% for 2 min, followed by two changes of 99% for 5 min each) and cleared twice in 99.9% xylene (University of Würzburg, Würzburg, Germany) for 5 min. Finally, specimens were mounted with DePeX mounting medium (10236276001, SERVA Electrophoresis GmbH, Heidelberg, Germany).

### 2.16 Elastic Stain

Elastic fibers were visualized using Resorcin‐Fuchsin‐Staining solution (MORPHISTO, 10354‐01, Offenbach, Germany) according to the protocol provided by the manufacturer.

### 2.17 Tissue Clearing

To visualize vascular network formation within the printed constructs, whole‐mount immunofluorescence staining was combined with ethyl cinnamate‐based tissue clearing as described previously [18]. Endothelial cells (ECs) and peri‐endothelial cells (PECs) were labeled using primary antibodies against CD31 and αSMA, respectively. Fluorescence was visualized with Cy2‐ and Cy3‐ conjugated secondary antibodies. Confocal imaging was performed as described above.

### 2.18 Monocyte Adhesion Assay

On culture day 7, vascular tubes were cut open under sterile conditions using a scalpel to expose the luminal surface. The opened tubes were flattened into rectangular shapes and placed in cell culture flasks with the luminal surface facing upward. Samples were incubated for 48 h in vascular growth medium supplemented with 15 ng/mL TNF‐α (Thermo Fisher, 300–01A, Waltham, MA, USA). Control samples were incubated in vascular growth medium without TNF‐α. After 48 h, TNF‐α was removed by washing all samples three times with cell culture medium for 5 min each.

THP‐1 monocytes (Cytion, 300356, Heidelberg, Germany) were cultured in RPMI‐1640 supplemented with 10% fetal bovine serum and 2 mM L‐glutamine (Capricorn, RPMI‐XA, Ebsdorfergrund, Germany; Capricorn, FBS‐11A; Thermo Fisher, 25 030 081) at 37°C and 5% CO_2_. Medium was renewed three times per week, and cell density was maintained between 1 × 10^5^ and 1 × 10^6^ cells/mL. Cultures exceeding this range were diluted accordingly.

Expanded monocytes were collected and pelleted by centrifugation at 900 rpm for 5 min. Cells were then labeled with calcein AM (CalAM) by resuspension in monocyte culture medium containing a 2 µm CalAM working solution and incubated for 20 min at 37°C. Following incubation, cells were washed three times with monocyte culture medium and centrifuged at 900 rpm for 5 min to remove excess CalAM. The monocyte pellet was resuspended in vascular growth medium. Labeled cells were imaged and counted using fluorescence microscopy to confirm viability and labeling efficiency.

Vascular growth medium containing fluorescently‐labeled monocytes at a concentration of 5 × 10^5^ cells/mL was added to all opened tubes, which were then incubated at 37°C and 5% CO_2_ for 1 h to allow monocyte adhesion. Subsequently, samples were washed with PBS three times for 15 min to remove non‐adherent monocytes. Nuclei were counterstained with DAPI (Merck, 2360276) for 10 min. After additional washing steps, samples were imaged from the luminal surface using confocal microscopy.

### 2.19 Perfusion of the Mother Vessel

We designed and fabricated a prototype perfusion chamber to mimic physiological flow conditions by enabling controlled perfusion through 3D‐bioprinted tubular structures. The chamber allows parallel perfusion of two tubular constructs within a shared chamber geometry, enabling simultaneous flow through two vessels in a defined and aligned orientation. The chamber accommodates the printed constructs within a rectangular compartment (22 mm × 22 mm × 8 mm; L × W × H) and is perfused via Luer‐lock ports after assembly. Sealing between the upper and lower half‐shells is achieved using a two‐component silicone (SF33, silicone fabric). The printed perfusion chamber is reproducible, readily customizable to experimental requirements, and biocompatible.

The upper and lower half‐shells that jointly form the perfusion chamber were designed using 3D CAD software (SolidWorks). The corresponding STL files were transferred to a 3D printer (Elegoo Saturn 4 Ultra, 16K) for fabrication. Both components were printed using a biocompatible resin based on BioMed Clear (LiQcreate, The Netherlands) and subsequently post‐cured under 405 nm UV light for 20 min. After printing, the components were removed from the build platform and washed in 96% ethanol (MEK, Nordbrand) to remove residual resin.

To generate a closed medium reservoir and enhance optical clarity, a 1‐mm glass plate was bonded to the top surface of the upper half‐shell and to the bottom surface of the lower half‐shell. The two half‐shells were then aligned and secured at the corners using small screws. Lateral interfaces were sealed with plastic Luer‐lock adapters (Borem; Luer‐lock to 1/8″ tubing). Phosphate‐buffered saline (PBS) was injected through one adapter using a syringe, and the fully assembled chamber was filled and incubated overnight to assess leakage. No leakage was observed.

Prior to assembly, all components of the perfusion setup were sterilized 3 × 30 min by immersion in 70% ethanol. The parts were subsequently removed from ethanol, rinsed twice in sterile PBS for 10 min each, air‐dried under a sterile bench for 1 h, and post‐cured under UV light for 15 min on each side. Following UV curing, day‐7 bioprinted tubular constructs were assembled into the perfusion chamber and perfused under slow pulsatile flow (1 mL/min) using oxygenated basal culture medium delivered by a peristaltic pump system (Golander B100, adjustable flow mode and rate; Golander GmbH, Bonn, Germany).

### 2.20 Statistical Analysis

The statistical analyses were performed using GraphPad Prism 11 (GraphPad Software). Data are presented as mean ± SD unless stated otherwise. Normal distribution of the data was assessed using a Shapiro‐Walk test (alpha = 0.5). Statistical significance was depicted as ^*^ *p* <0.05, ^**^ *p* <0.01, ^***^ *p* <0.001, ^****^ *p* <0.0001.

#### 2.20.1 Compressive Mechanical Evaluation of Hydrogels

Sample size n = 3, normal distribution confirmed. One‐way ANOVA followed by Tukey's multiple comparisons test was performed to evaluate significant differences between hydrogels. The corresponding p‐values were: GelMA vs. GelMA+Col I: 0.9976, GelMA vs. FGX: 0.9866, GelMA vs. FGXC: 0.0019, GelMA+Col I vs. FGX: 0.9535, GelMA+Col I vs. FGXC: 0.0015, FGX vs. FGXC: 0.0028.

#### 2.20.2 Cell Viability in FGXC

Sample size n = 4, normal distribution confirmed. One‐way ANOVA followed by Tukey's multiple comparisons test was performed to evaluate significant differences over time. The corresponding p‐values were: D1 vs. D3: 0.5093, D1 vs. D7: 0.1913, D3 vs. D7: 0.8001.

#### 2.20.3 Vascular Wall Thickness

Sample size n = 3, normal distribution confirmed. One‐way ANOVA followed by Tukey's multiple comparisons test was performed to evaluate significant differences over time compared to day 4. The corresponding p‐values were: D4 vs. D7: <0.0001, D4 vs. D9: <0.0001, D4 vs. D13: <0.0001.

#### 2.20.4 Lumen Diameter

Sample size n = 4, normal distribution confirmed. One‐way ANOVA followed by Tukey's multiple comparisons test was performed to evaluate significant differences over time compared to day 4. The corresponding p‐values were: D4 vs. D7: 0.0028, D4 vs. D9: <0.0001, D4 vs. D11: <0.0001, D4 vs. D13: <0.0001.

#### 2.20.5 Mesodermal Tissue Thickness

Sample size n = 7, normal distribution confirmed. One‐way ANOVA followed by Tukey's multiple comparisons test was performed to evaluate significant differences over time compared to day 4. The corresponding p‐values were: D4 vs. D7: 0.3459, D4 vs. D9: <0.0001, D4 vs. D11: <0.0001, D4 vs. D13: <0.0001.

#### 2.20.6 Endothelial Branch Length

Data are presented as individual data points (branch length) from different samples (at least 750 points per sample), sample size of n = 3. Since the data were not distributed, the non‐parametric Kruskal‐Wallis test followed by Dunn's multiple‐comparison test was used to evaluate significant differences over time. The corresponding p‐values were: D3 vs. D7: <0.0001, D3 vs. D10: <0.0001, D7 vs. D10: > 0.9999.

#### 2.20.7 Vessel Surface Area

Sample size n = 3. Since the data were normally distributed, the non‐parametric Kruskal–Wallis test followed by Dunn's multiple‐comparison test was used to evaluate significant differences over time. The corresponding p‐values were: D3 vs. D7: 0.0373, D3 vs. D10: 0.7907, D7 vs. D10: 0.7907.

#### 2.20.8 Luminal Surface Coverage

Sample size n = 3, normal distribution confirmed. One‐way ANOVA followed by Tukey's multiple comparisons test was performed to evaluate significant differences over time. The corresponding p‐values were: D3 vs. D7: 0.1150, D3 vs. D10: <0.0001, D7 vs. D10: 0.0003.

#### 2.20.9 Monocyte Adhesion

Sample size n = 3, normal distribution confirmed. An unpaired t‐test with Welch's correction (two‐tailed) was used to evaluate significant differences (*p*‐value: 0.0218).

## 3 Results and Discussion

**FIGURE 1:**
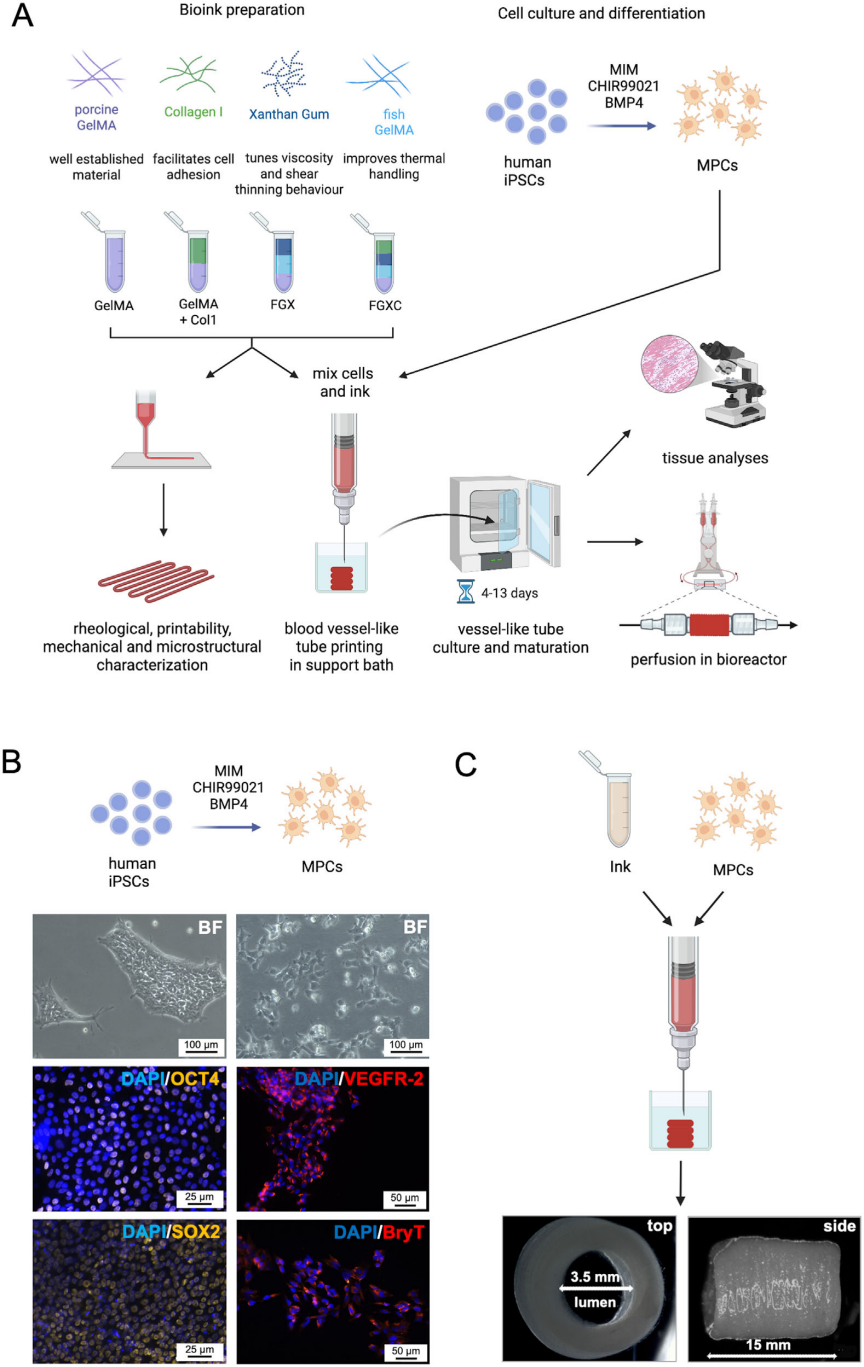
Workflow for generating hiMPCs from hiPSCs and bioprinting hiMPC‐laden hydrogels to produce vascularized tubular structures. (A) Schematic overview of the experimental workflow depicting bioink composition, cell differentiation, printing of tubular constructs into a support bath, and subsequent culture and analyses. (B) Differentiation of hiPSCs into mesodermal progenitor cells (MPCs). During 2D differentiation, cells change morphology from small, round hiPSCs growing in colonies to more flattened MPCs with a mesenchymal appearance. hiPSCs express the pluripotency markers OCT4 and SOX2, whereas MPCs express mesodermal markers VEGFR‐2 and Brachyury (BryT). (C) Schematic depiction of the embedding bioprinting process using a support bath and representative images of printed tubular constructs shown in top and side views (lumen diameter: 3.5 mm; tube length: 15 mm). The Schematics in Figure 1 Were Created in BioRender. Wörsdörfer, P. (2026) https://www.BioRender.com/5o6e1m0.

Human induced pluripotent stem cells (hiPSCs; SOX2^+^ and OCT4^+^) (Figure 1B) were differentiated into hiMPCs using a previously established 2D culture protocol developed in our laboratory [14] (Figure 1A). Immunofluorescence analyses for the mesodermal progenitor markers VEGFR‐2 and Brachyury confirmed successful differentiation (Figure 1B).

We chose hiMPCs as the cell source for bioink formulation, as they can differentiate into all cell types of the vascular wall and form hierarchically organized, perfusable vessel‐like structures following extrusion bioprinting [5].

For each experimental batch, hiMPCs were freshly induced shortly before printing. The bioink was prepared by evenly suspending the cells at a density of 2 × 10^7^ cells per milliliter of hydrogel (Figure 1A,C). Throughout this study, embedding bioprinting was employed to fabricate large‐scale tubular structures by depositing the bioink into an XG‐based support bath (Figure 1A,C). Following printing, the structures were stabilized by UV crosslinking and subsequently cultured under static conditions for 3–13 days (Figure 1A).

### 3.1 Bioink Characterization and Optimization

To develop a bioink that combines both reliable printability and the capacity to support vascular morphogenesis, several candidate hydrogels were initially evaluated. Preliminary experiments with alginate‐ and fibrin‐based matrices revealed substantial limitations, as neither material allowed the cells to undergo morphogenetic processes required for vessel wall formation.

We therefore focused on gelatin methacryloyl (GelMA) as a versatile base matrix with tunable physical and biological properties. However, porcine GelMA alone was unable to provide the thermal stability, rheological control and mechanical robustness required for the fabrication of large “mother vessel” constructs. To overcome these limitations, we systematically optimized the formulation by incorporating defined additives with complementary functions: Col I to enhance cell adhesion and mimic native extracellular matrix composition, piscine GelMA to improve thermal handling during the printing process due to its lower gelling temperature, xanthan gum to tune viscosity and enable shear‐thinning behavior and fibrillar Col I to reinforce the mechanical stability and homogeneity of the matrix (Figure 1A).

**FIGURE 2:**
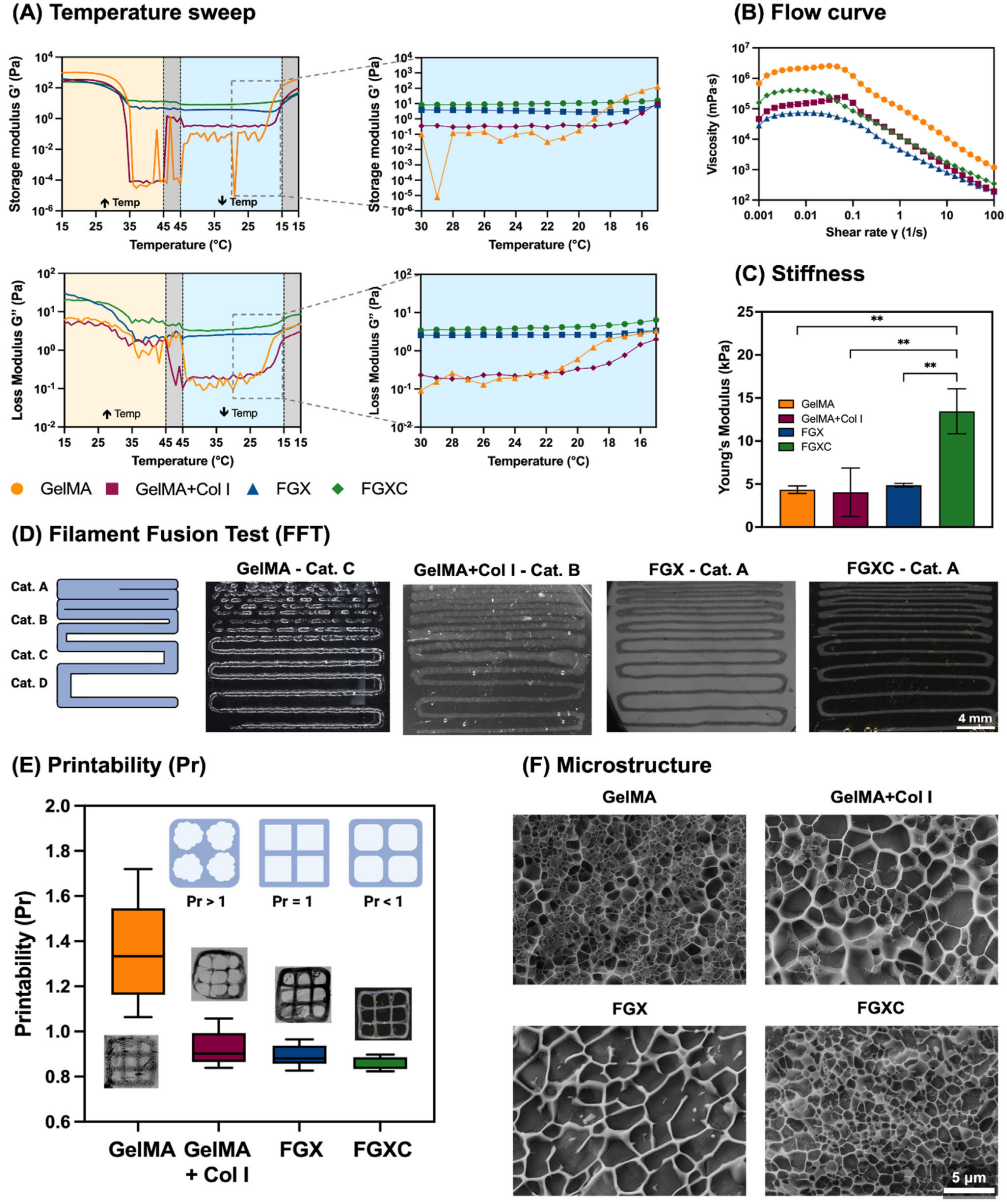
Rheological, printability, mechanical, and microstructural characterization of the tested hydrogel formulations. (A) Temperature sweep analysis showing storage modulus (G′) and loss modulus (G″) as a function of temperature prior to crosslinking. (B) Flow curves depicting viscosity as a function of shear rate for non‐crosslinked bioinks. (C) Compressive Young's modulus of crosslinked hydrogels. Normal distribution of the data confirmed by Shapiro‐Walk test. One‐way ANOVA and Tukey's multiple comparisons test were performed to evaluate significant differences over time (n = 3). Statistical significance depicted as ^*^p <0.05, ^**^p <0.01, ^***^p <0.001, ^****^p <0.0001 (D) Filament fusion test (FFT) used to assess print resolution and categorize bioinks according to Lamberger et al. (2024). (E) Quantitative printability index (Pr) analysis based on pore geometry according to Ouyang et al. (2016). (F) Microstructural analysis of crosslinked hydrogels by cryo‐SEM.

Four GelMA‐based blends of increasing compositional complexity were compared: (1) GelMA without any additives as baseline, (2) GelMA + Col I, (3) FGX (piscine + porcine GelMA + xanthan gum), and (4) FGXC (FGX + fibrillar Col I). Each formulation was systematically characterized for rheological behavior, printability, mechanical performance, and microstructural organization (Figure 2). Rheological measurements revealed distinct viscoelastic profiles of the tested formulations. Temperature‐sweep tests (15°C‐45°C) showed that both FGX and FGXC maintained stable storage (G′) and loss (G″) moduli across the full range, indicating temperature‐stable viscoelastic behavior and suitability for bioprinting under room temperature (RT) conditions. In contrast, GelMA and GelMA + Col I exhibited sharp thermal transitions around 30°C, leading to unstable flow behavior and reduced process reliability (Figure 2A). Shear‐rate sweeps (0.001–100/s) further demonstrated pronounced shear‐thinning for FGX and FGXC, enabling consistent extrusion at low pressures (< 15 kPa) (Figure 2B).

Printability was initially determined using the filament‐fusion test (FFT) (Figure 2D) and a printability index (Pr) (Figure 2E). GelMA (Pr = 1.35 ± 0.22, Cat C) and GelMA + Col I (Pr = 0.92 ± 0.07, Cat B) exhibited irregular pore geometry and intermittent filament flow, occasionally due to collagen aggregation. In contrast, FGX (Pr = 0.89 ± 0.05, Cat A) and FGXC (Pr = 0.87 ± 0.03, Cat A) yielded well‐defined, continuous filaments with uniform deposition and minimal over‐ or under‐extrusion (Figure 2D).

**FIGURE 3:**
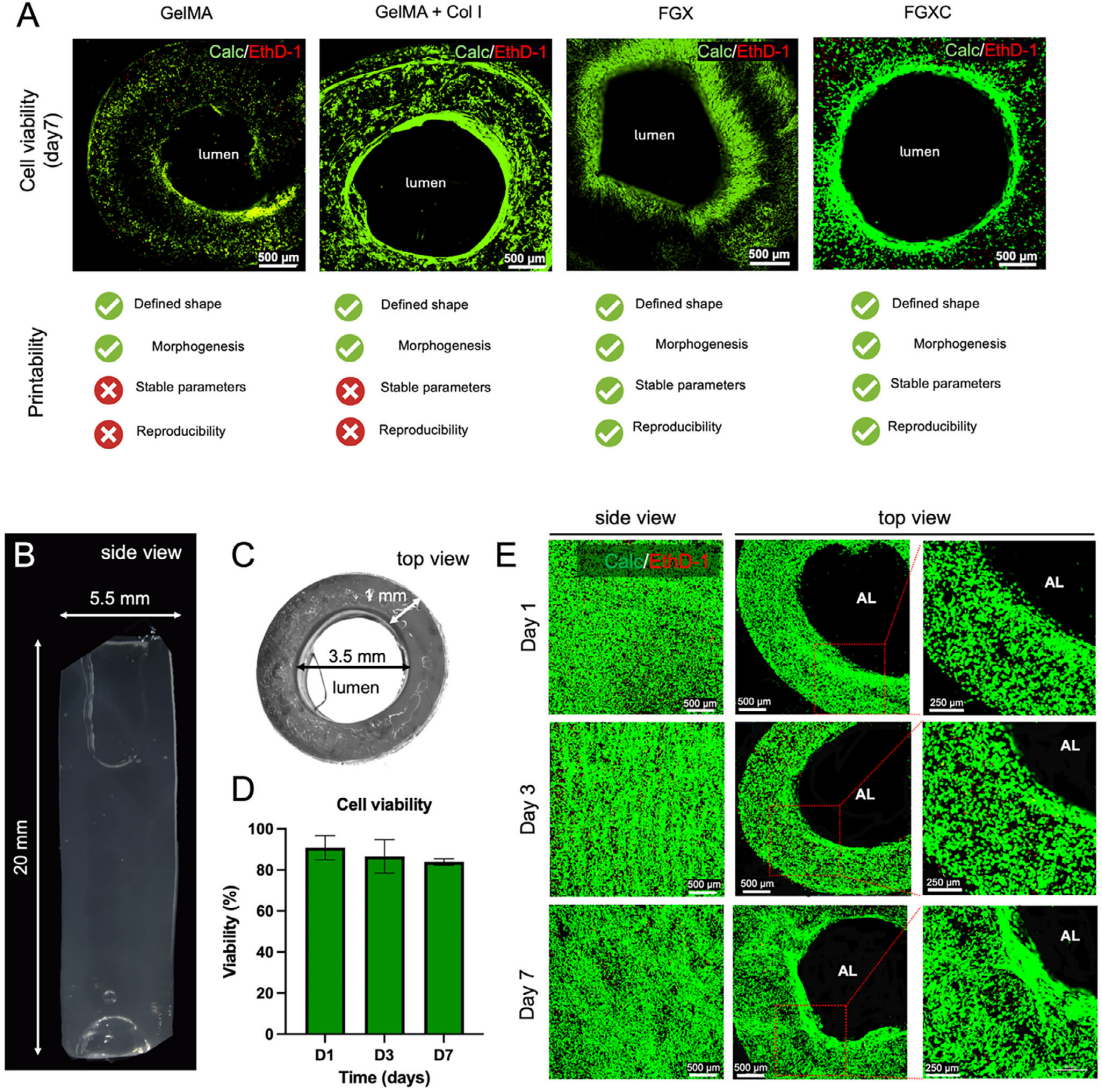
Printability of hiMPC‐laden bioinks and cell viability over time in printed constructs using the final bioink formulation (FGXC). (A) Representative images of printed tubular structures at day 7 of maturation generated using four tested bioinks (GelMA, GelMA + Collagen I, FGX, and FGXC). Cell viability was assessed using a calcein/EthD‐1 live/dead assay. An evaluation of printing outcome with respect to tube geometry, tissue morphogenesis during culture, parameter stability, and reproducibility is shown. (B) Representative side‐view image of a printed tube (tube length: 20 mm; tube width: 5.5 mm). (C) Representative top‐view image of a printed tube (lumen diameter: 3.5 mm; printed wall thickness: 1 mm). (D) Quantification of cell viability at days 1, 3, and 7 after printing as determined by the calcein/EthD‐1 live/dead assay. Normal distribution of the data confirmed by Shapiro–Walk test. One‐way ANOVA and Tukey's multiple comparisons test were performed to evaluate significant differences over time (n = 4). Statistical significance depicted as ^*^p <0.05, ^**^p <0.01, ^***^p <0.001, ^****^p <0.0001. (E) Representative images corresponding to the quantitative analyses shown in (D).

Mechanical compression testing showed that the combined addition of xanthan gum and fibrillar Col I markedly increased the mechanical stability of the hydrogels. The corresponding Young's moduli were 4.3 ± 0.4 kPa (GelMA), 5.2 ± 0.5 kPa (GelMA + Col I), 4.9 ± 0.2 kPa (FGX) and 13.4 ± 2.6 kPa (FGXC). Thereby, FGXC was in the mechanical range of soft connective tissue [19] (Figure 2C). Cryo‐SEM analysis further confirmed distinct microstructural differences among the tested hydrogels. FGXC exhibited a dense, homogeneous fibrillar network with uniformly distributed pores, whereas GelMA, GelMA + Col I and FGX were more heterogeneous with variable pore size and network density, consistent with their lower printability index and stiffness (Figure 2F). Hence, the incorporation of cold‐water‐fish‐derived GelMA improved thermal stability and printability, while fibrillar Col I enhanced mechanical strength and homogeneity of matrix organization. Together, these findings identify FGXC as the optimal bioink formulation, as it combines temperature stability, shear‐thinning rheology and mechanical robustness with high print fidelity (Figure 3A).

### 3.2 Embedded 3D Bioprinting in an XG‐HA Support Bath

For the direct bioprinting of free‐standing tubular constructs, cell‐laden bioinks were extruded into a viscoelastic support bath composed of xanthan and hyaluronic acid in HEPES buffer (Figure 1C). The support bath provided transient mechanical stabilization during extrusion. This enabled the use of soft, low‐viscosity hydrogels that would otherwise collapse under their own weight. Moreover, the viscoelastic behavior of the XG‐HA matrix created gentle shear conditions that preserved cell integrity and supported morphogenetic cell behavior during and after printing. The support bath exhibited self‐healing, fluid‐like behavior under applied stress and pronounced shear‐thinning, as confirmed by stress‐recovery, flow‐curve and frequency‐sweep analyses (Figure S1C). Thereby, the support bath not only ensured temporary mechanical support during the printing process but also enabled the gentle release of the vascular constructs after crosslinking, maintaining structural integrity [20, 21, 22, 23, 24].

Using the optimized FGXC formulation, tubular structures were printed at RT under low extrusion pressure (< 20 kPa) through 20–22 G needles (20 G for FGXC). Constructs were photopolymerized (405 nm, 3 min; 54 mW/cm^2^) and then gently released from the support bath by rinsing. This removed residual XG without affecting the printed geometry and resulted in self‐standing tubes with interlayer fusion, homogeneous wall structure and tunable dimensions (up to 1.5–2 cm length, 3.5–4.5 mm inner lumen diameter) (Figures 1C and 3B,C).

In parallel, the biological evaluation of hiMPC‐laden constructs revealed that all formulations maintained high cell viability, with stable tube geometry and the formation of a continuous cell–cell layer around the artificial lumen (Figure 3A). To improve cell adhesion and mimic the extracellular matrix composition of native vascular tissue, Col I was incorporated into GelMA. Both GelMA and GelMA + Col I supported early endothelial alignment. However, most cells accumulated along the tube borders, reflecting heterogeneous matrix density and uneven cell distribution within the hydrogel. To further enhance temperature stability and extrusion consistency, cold‐water‐fish‐derived GelMA was introduced, and xanthan gum was added to tune viscosity and promote shear‐thinning behavior during printing. The FGX formulation improved cell retention and uniform distribution throughout the tube wall and promoted cord‐like endothelial structures. Finally, supplementing the FGX blend with fibrillar Col I provided additional mechanical reinforcement and improved matrix homogeneity. The optimized FGXC bioink combined these features, enabling homogeneous cell localization, rapid elongation and the formation of interconnected CD31^+^/αSMA^+^ vascular‐like networks within seven days of culture. Over time, the gradual release of XG increased matrix microporosity (Figure S2) and facilitated directed cell migration and network formation. For that reason, the FGXC bioink was used for all the following experiments.

### 3.3 Vascular Morphogenesis and Vessel Wall Formation

**FIGURE 4:**
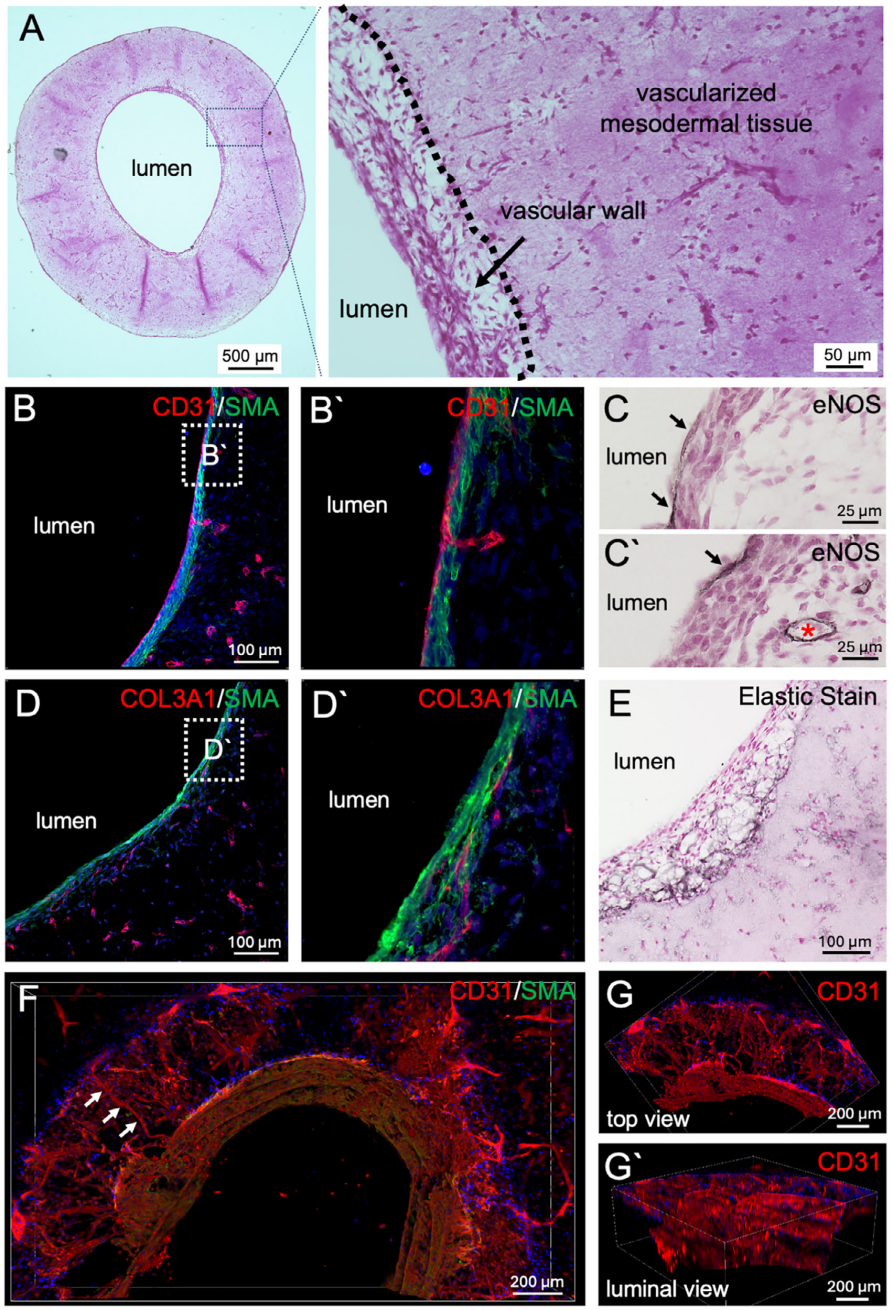
Histological analyses of printed tubes generated from hiMPCs using the FGXC bioink at day 7 of differentiation. (A) H&E staining of paraffin sections from printed tubes at day 7 of culture. The left image shows an overview of the entire tube, while the right image displays a higher‐magnification view focusing on the developing vascular wall lining the tube lumen. Distinct vessel wall layers are detectable. (B–B′) Immunofluorescence staining for SMA (smooth muscle cells) and CD31 (endothelial cells). CD31^+^ endothelial cells line the lumen, forming the intima. SMA^+^ smooth muscle cells form a continuous circumferential layer beneath the intima, resembling a tunica media. Endothelial sprouts are detectable throughout the construct. (B′) shows a higher‐magnification view highlighting an endothelial sprout extending from the intima into the vessel wall. (C–C′) Immunohistochemical detection of eNOS. eNOS^+^ endothelial cells are present in the intima (black arrows) and in smaller vessels within the vascular wall (red asterisk). (D–D′) Co‐immunofluorescence analysis for COL3A1 and SMA. COL3A1 deposition is detected within the vascular wall in close proximity to the SMA^+^ tunica media and along endothelial sprouts. (E) Elastin staining revealing elastic fiber deposition within the developing vascular wall. (F) Three‐dimensional reconstruction of tissue‐cleared samples stained for CD31 and SMA illustrating the 3D architecture of the printed tubes, including endothelial cords and capillary‐like structures. Capillary‐like structures spanning the vessel wall from lumen to periphery of the whole construct are indicated by white arrows. (G–G′) Higher‐magnification views of (F) from the top and from the luminal side, highlighting the endothelial lining of the intima from two different angles.

**FIGURE 5:**
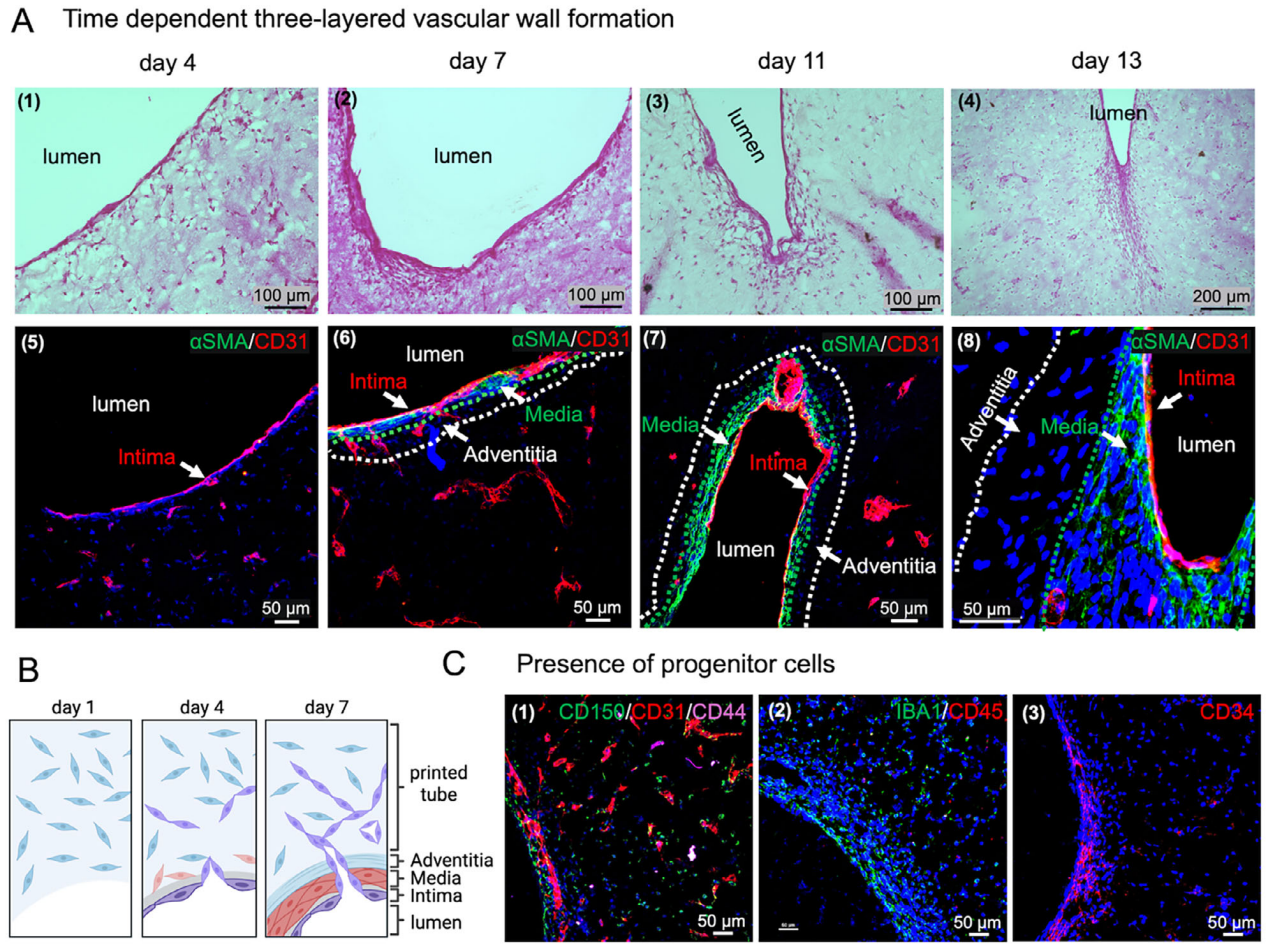
Evaluation of tissue morphogenesis in hiMPC‐laden FGXC‐printed tubular constructs over two weeks of culture. (A1–A4) Representative H&E stainings at days 4, 7, 11, and 13 of differentiation, demonstrating progressive vessel wall organization and maturation. (A5–A6) Co‐immunofluorescence analyses for CD31 and SMA at days 4, 7, 11, and 13, revealing the gradual formation of a three‐layered vascular wall. The CD31^+^ endothelial innermost layer corresponds to the intima, the circumferential SMA^+^ layer represents the tunica media, and the outer SMA^−^ layer, containing endothelial sprouts and capillary‐like structures, resembles the tunica adventitia. (B) Schematic overview summarizing the temporal progression of vessel wall development shown in (A). (C) Detection of CD150^+^, CD44^+^, and CD34^+^ putative progenitor cells within the vascular wall. IBA1^+^ macrophage‐like cells are also present within the constructs. The schematic in Figure 5B was created using BioRender (Wörsdörfer, P., 2026; https://BioRender.com/5m856if).

Following confirmation of high post‐printing viability (Figure 3D–E), vascular morphogenesis and vessel wall organization were examined within the bioprinted FGXC “mother vessels” (Figures 4 and 5).

At day 7, we observed that the luminal surface of the printed tube was lined by a three‐layered vascular wall of approximately 150 µm thickness (Figure 4A). The main part of the printed construct consisted of a loose, vascularized mesodermal tissue (Figure 4A). Immunofluorescence analyses revealed a CD31^+^ endothelial lining of the lumen of the printed tube (Figure 4B,B′). In addition, CD31^+^ endothelial sprouts and capillary‐like structures were detectable within the mesodermal tissue (Figure 4B). Connections between the vascular network within the mesodermal wall of the tube and the lumen could also be detected (Figure 4B′). The CD31^+^ cells within the printed vessel‐like tube displayed eNOS expression (Figure 4C–C'), further confirming their endothelial identity.

Beneath the endothelial lining of the lumen, representing the intimal compartment of the vessel wall, 3–4 SMA^+^ cell layers were detected, forming a tunica media‐like smooth muscle ring (Figure 4B,B′,D,D′). Within this medial vessel wall compartment, as well as along endothelial sprouts, COL3A1 deposition was observed (Figure 4D–D′). Moreover, Elastin staining revealed deposition of elastic fibers within the vessel wall (Figure 4E). This indicates early basement membrane‐like formation and extracellular matrix remodeling conducive to the development of a multilayered vessel wall [25]; Gross et al., 2021; Kanie et al., 2012; Mak & Mei, 2017). The SMA^+^ media‐like tissue as well as the intima‐like endothelial lining of the lumen were further demonstrated using tissue‐clearing analyses (Figure 4F–G′ and Figure S3, Video). These analyses also showed the microvascular network within the mesodermal tissue and revealed vascular sprouts originating from the lumen and spanning the complete wall of the printed tube (Figure 4F, white arrows).

To assess the timeline of vascular development, constructs were cultured under static conditions for up to 13 days and analyzed histologically at days 4, 7, 11, and 13. At day 4, scattered CD31^+^ endothelial clusters as well as focal endothelial lining of the vascular lumen became visible, indicating the onset of endothelial differentiation and partial lining of the luminal surface by endothelial cells (Figure 5A1 and A5). By day 7, CD31^+^ cells elongated and aligned circumferentially along the artificial lumen (Figure 5A2, A6). This was accompanied by emerging cord‐like vascular structures within the surrounding mesoderm‐like tissue. By day 7, the cord‐like structures evolved into an interconnected CD31^+^ capillary‐like network with branching and sprouting morphologies, both characteristic of vasculogenic and angiogenic processes (Figure 5A2, A6,B). These findings demonstrate that bioprinted hiMPCs rapidly differentiate into endothelial lineages that not only line the luminal surface of the artificial tube but also self‐organize into vascular‐like networks, thereby recapitulating key steps of developmental vessel formation in vitro.

Consistent with these observations, H&E and immunofluorescence analyses of day 7 constructs revealed an early multilayered vessel wall‐like formation lining the lumen of the printed tube along its entire circumference (Figures 4A and 5A). The CD31^+^ intimal layer lining the lumen was encased by a circumferential layer composed of multiple sheets of αSMA^+^ smooth muscle cells (SMCs), forming a nascent tunica media (Figure 5A2,A6–A8). Moreover, a third layer of loose connective tissue was observed between the SMA^+^ media and the printed tube (Figure 5A6–A8,B, day 7). The progression of the vessel wall formation, including the development of the distinct layers such as intima, media, and adventitia is summerized by the schematic overview (Figure 5B). Furthermore, the newly formed vessel wall, particularly its adventitial layer, harbored CD150^+^, CD44^+^, CD45^+^, and CD34^+^ hematopoietic and vascular progenitors, closely mimicking the adventitial layer of native blood vessels (Figure 5C1–3) [2, 26, 27, 28, 29, 30]. The three‐layered vessel wall organization further matured until day 11, with the media layer becoming more prominent (Figure 5A3 and A7). By day 13, H&E and immunofluorescence staining showed narrowing of the artificial lumen (Figure 5A4 and A8) and reorganization of the vessel wall, leading to collapse of the printed tube and loss of the well‐organized three‐layered wall lining the mother vessel lumen.

**FIGURE 6:**
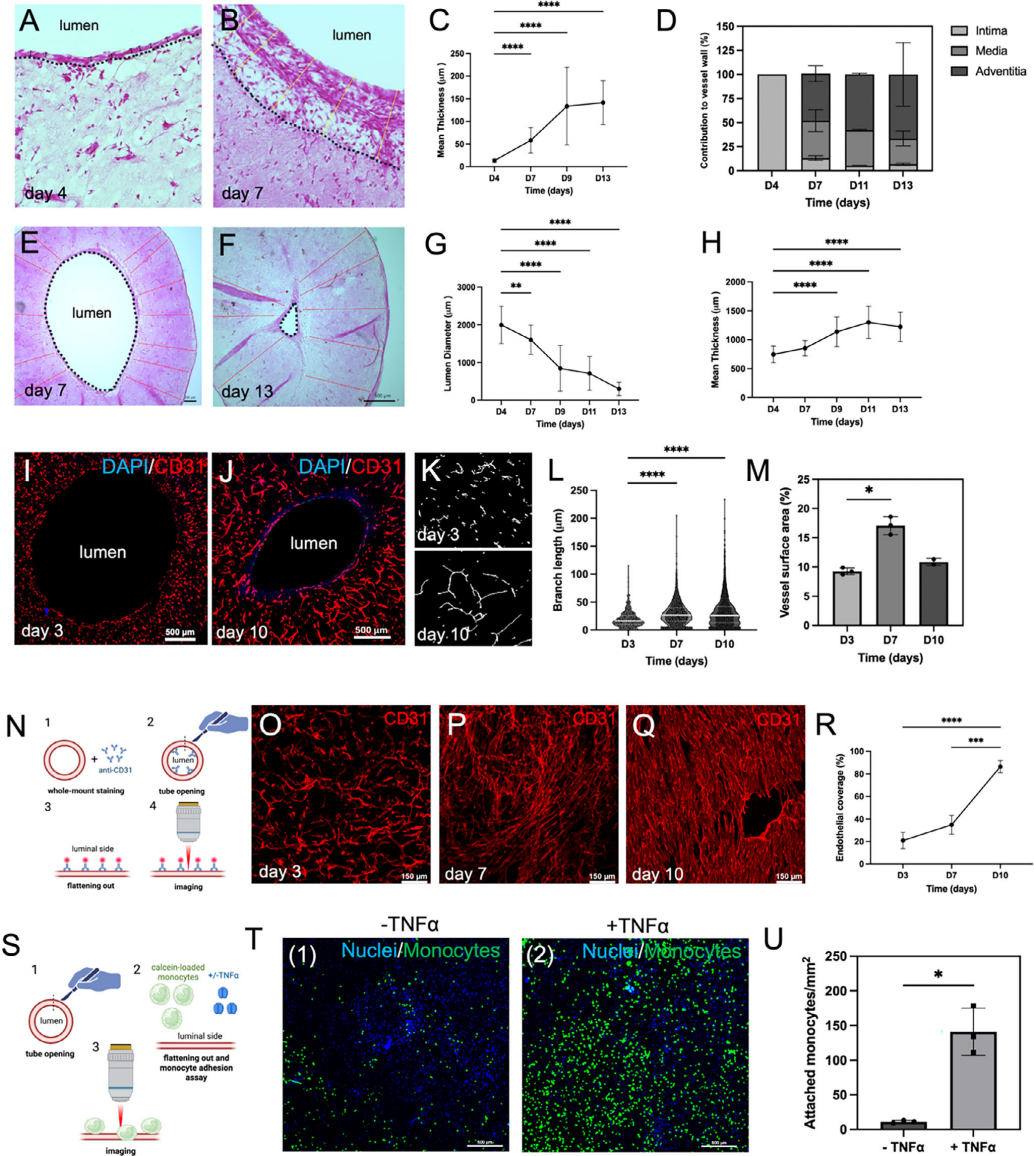
Quantification of vessel wall dimensions, vascular network formation, endothelial coverage, and monocyte adhesion. (A–B) H&E stainings of the vascular wall at days 4 and 7 of culture. Black dotted lines mark the border between the vascular wall and surrounding mesodermal tissue. Increasing wall thickness and differentiation toward a three‐layered architecture are evident. (C) Quantification of vascular wall thickness from day 4 to day 13, showing a significant increase to approximately 150 µm by day 13. Normal distribution of the data confirmed by Shapiro‐Walk test. One‐way ANOVA and Tukey's multiple comparisons test were performed to evaluate significant differences over time. The graph depicts comparisons to day 4 (n = 3). (D) Relative contributions of intima, media, and adventitia to the vessel wall over time, expressed as percentages. (E,F) H&E stainings highlighting the lumen of the printed tube (outlined by black dotted lines), demonstrating progressive lumen narrowing.(G) Quantification of lumen diameter showing gradual and significant reduction during culture. Normal distribution of the data confirmed by Shapiro–Walk test. One‐way ANOVA and Tukey's multiple comparisons test were performed to evaluate significant differences over time. The graph depicts comparisons to day 4 (n = 4). (H) Quantification of mesodermal tissue thickness surrounding the printed tube, which initially increases and stabilizes from day 9 onward. Normal distribution of the data confirmed by Shapiro‐Walk test. One‐way ANOVA and Tukey's multiple comparisons test were performed to evaluate significant differences over time. The graph depicts comparisons to day 4 (n = 7). (I,J) CD31 immunofluorescence staining visualizing endothelial networks within the mesodermal tissue at days 3 and 10. Samples: 300 µm vibrotome sections. (K) Higher‐magnification view showing skeletonization of the endothelial network used for quantitative analyses. (L) Quantification of endothelial branch length over time, revealing a significant increase during culture. Non‐parametric Kruskal‐Wallis test and Dunn's multiple comparisons test were performed to evaluate significant differences over time. The graph depicts the distribution of the length of individual branches from multiple samples (n = 3). (M) Quantification of the CD31^+^ area within the mesodermal tissue, which increases from day 3 to day 7 and subsequently decreases, indicating vascular network maturation and remodeling. Non‐parametric Kruskal‐Wallis test and Dunn's multiple comparisons test were performed to evaluate significant differences over time (n = 3). (N) Schematic workflow for analysis of endothelial coverage of the intimal layer. (O–Q) Representative CD31 immunofluorescence images showing endothelial coverage of the luminal surface at days 3, 7, and 10. (R) Quantification of luminal surface coverage by CD31^+^ endothelial cells over time, reaching nearly 90% by day 10. Normal distribution of the data confirmed by Shapiro‐Walk test. One‐way ANOVA and Tukey's multiple comparisons test were performed to evaluate significant differences over time (n = 3). (S) Schematic workflow of the monocyte adhesion assay. (T) Representative images of monocyte adhesion without (1) and with TNFα stimulation (2), showing increased adhesion following inflammatory activation. (U) Quantification of adhered monocytes. Normal distribution of the data confirmed by Shapiro‐Walk test. Unpaired t‐test with Welch's was performed to evaluate significant differences (n = 3). Statistical significance depicted as ^*^p <0.05, ^**^p <0.01, ^***^p <0.001, ^****^p <0.0001. The schematics in Figure 6N,S were created in BioRender. Wörsdörfer, P. (2026) https://BioRender.com/z44lxop and https://BioRender.com/9qt6r39.

We next quantified the dimensions of the vascular wall and its changes from day 3 to day 13 (Figure 6A–C). We observed an increase in wall thickness from initially a few micrometers to approximately 150 µm by day 13 (Figure 6C). At day 3, only a thin intima‐like lining of the tube was apparent (Figure 6A). By day 7, the characteristic three‐layered structure of the vessel wall, including intima, media, and adventitia, became visible (Figure 6B). The relative contributions of these layers changed over time, as shown in Figure 6D.

We also measured lumen diameter over time and observed that the initially 3.5‐mm‐wide lumen after printing progressively shrank during culture and nearly collapsed into a narrow channel by day 13. The vascularized mesodermal compartment surrounding the printed tube initially increased in size until day 11 and then stabilized. To assess vascular network formation within the mesodermal tissue, we stained for CD31 (Figure 6I,J), skeletonized the CD31^+^ networks (Figure 6K), and quantified endothelial branch length (Figure 6L) and CD31^+^ area coverage (Figure 6M). Branch lengths significantly increased over the culture period. The area covered by CD31^+^ cells increased from day 3 to day 7 and subsequently decreased by day 10, indicating vascular network maturation and remodeling.

We also quantified endothelial coverage of the luminal surface. For this, printed tubes were whole‐mount stained for CD31 at days 3, 7, and 10, cut open, flattened with the luminal surface facing upward, and imaged (Figure 6N) using confocal laser scanning microscopy. We observed a progressive increase in endothelial coverage from day 3 to day 10 (Figure 6O–R), reaching nearly 90% luminal coverage by day 10.

Finally, we performed a monocyte adhesion assay to functionally assess the endothelial lining of the mother vessel lumen. Tubes at day 10 were cut open and flattened with the luminal side facing upward (Figure 6S). The endothelium was treated with TNFα for several hours, followed by the addition of green fluorescent monocytes. After washing, monocyte adhesion was quantified in TNFα‐treated and untreated control samples. We observed a significantly increased monocyte adhesion following TNFα treatment (Figure 6T–U), indicating an in vivo‐like endothelial functionality with respect to inflammatory leukocyte recruitment.

### 3.4 Integration of Prevascularized Mesodermal Organoids With Bioprinted “Mother Vessels”

**FIGURE 7:**
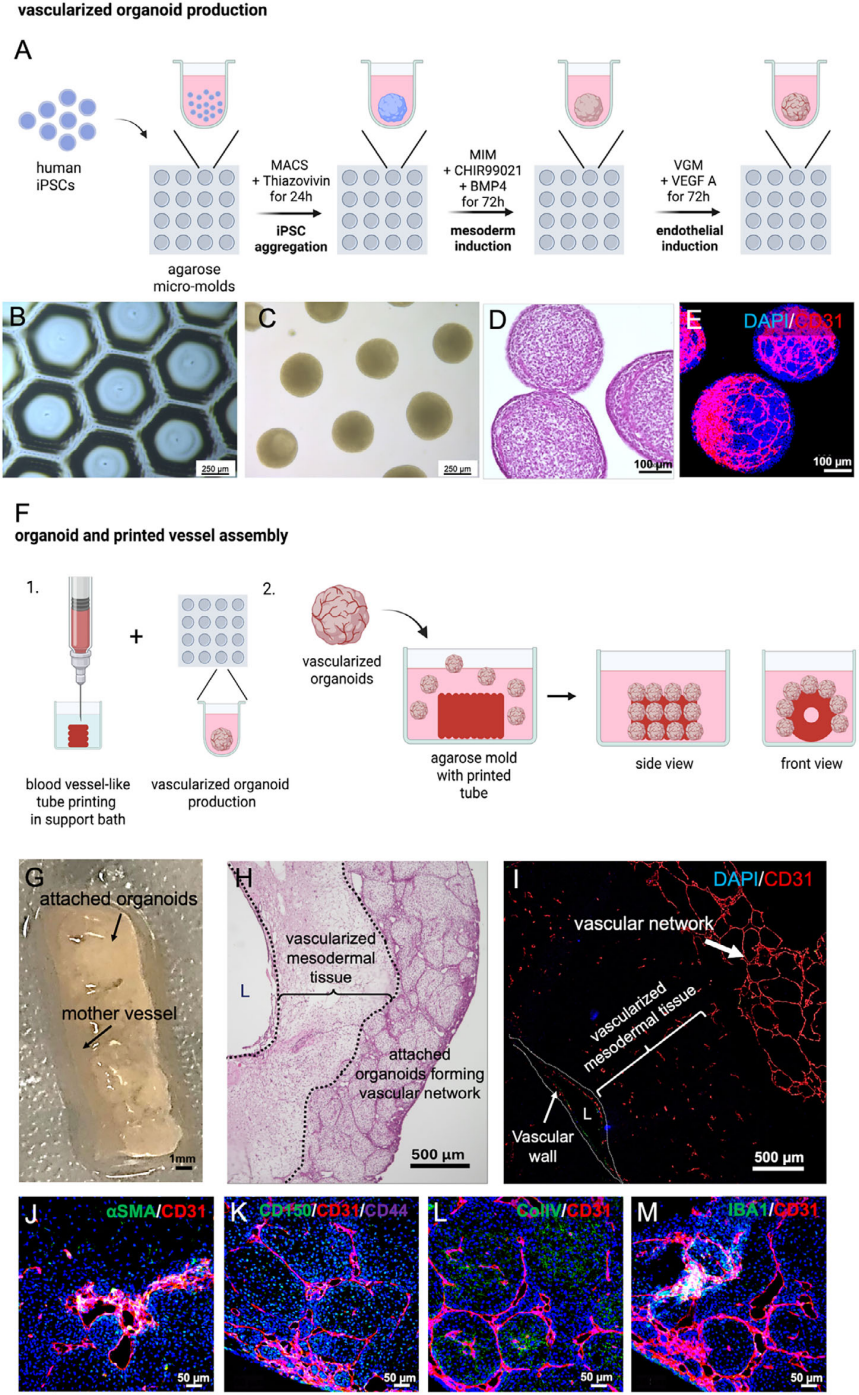
Assembly of biofabricated vascular constructs with pre‐vascularized mesodermal organoids. (A) Schematic illustration of the workflow for generating vascularized mesodermal organoids. (B) Agarose microwell molds used for organoid culture prior to cell seeding. (C) Representative images of organoids at culture day 7 showing uniform size and morphology. (D) H&E staining of sections from vascularized mesodermal organoids. (E) Whole‐mount CD31 immunofluorescence staining combined with tissue clearing highlights endothelial networks within organoids. (F) Schematic workflow for assembly of printed, cell‐laden tubular constructs with mesodermal organoids. (G) Image of a printed tube with adherent organoids. (H) H&E staining of paraffin sections through printed tubes with adherent organoids, demonstrating formation of a continuous tissue layer integrating with the printed tube. (I) CD31 immunofluorescence analysis showing distribution of capillary‐like structures and endothelial sprouts within the assembled construct. (J) Association of organoid‐derived vasculature with SMA^+^ peri‐endothelial cells. (K) Presence of CD44^+^ and CD150^+^ putative progenitor cells within the organoid layer. (L) Collagen IV deposition around capillary‐like structures within the organoid‐derived tissue. (M) Detection of IBA1^+^ macrophage‐like cells within the organoid layer. The Schematics in Figure 7A and F Were Created in BioRender. Wörsdörfer, P. (2026) http://www.BioRender.com/zde9xwa.

To extend vascularization beyond the printed tube wall, prevascularized mesodermal organoids ([31] were generated and co‐cultured with bioprinted FGXC “mother vessels” (Figure 7A). Organoids were produced from hiPSCs using custom‐made agarose microwell molds (Figure 7B). Owing to this standardized production process, organoids displayed highly uniform size (Figure 7C) and histological appearance (Figure 7D). Whole‐mount immunofluorescence analyses confirmed the presence of a branched CD31^+^ endothelial network within the organoids (Figure 7E).

Approximately 750 organoids were subsequently co‐cultured with a 4‐day‐old bioprinted “mother vessel” for three days (Figure 7F). The organoids not only adhered to the outer surface of the printed tubes but also gradually fused with each other and with the vessel wall (Figure 7G,H), forming a continuous tissue‐like covering of the “mother vessel” (Figure 7H).

Vascular networks originating from adjacent organoids fused with one another (Figure 7I) and additionally exhibited spatial continuity with the vascular network inside the mesoderm‐like tissue compartment of the bioprinted “mother vessel,” indicating the onset of structural integration (Figure 7I). Histological and immunofluorescence analyses revealed vascular organization and maturation within the fused constructs. CD31^+^ endothelial cells and αSMA^+^ mural cells were distributed throughout the organoid‐derived layer, forming vessel‐like structures comparable to those within the printed “mother vessel” (Figure 7J,K). Collagen IV deposition along vessel walls (Figure 7L) indicated early basement membrane formation and vascular maturation. Furthermore, the presence of Iba1^+^ macrophage‐like cells (Figure 7M) suggested the emergence of immune cell populations that are known to contribute to vascular remodeling in vivo.

As a proof of concept, perfusability of the “mother vessel” was evaluated in a bioreactor using pulsatile flow generated by a pump connected to the perfusion chamber. The mother vessel was clamped at both ends to the inlet and outlet tubing of the reactor (Figure S4A, Video). Basal medium was perfused through the system as clearly visible at higher magnification in the video recording (Figure S4B, Video). Importantly, no detectable leakage along the length of the mother vessel was observed. Only a minor amount of fluid entered the chamber at the clamped inflow end (Figure S4C, Video), indicating high stability and tightness of the mother vessel wall and supporting its suitability for perfusion.

## 4 Conclusions

In this study, we successfully established the bioprinting of centimeter‐scale “mother vessel” constructs using hiMPCs and a newly optimized bioink composed of piscine GelMA, porcine GelMA, xanthan gum, and fibrillar Col I. This formulation provided both printability and biological compatibility enabling the single‐step generation of centimeter‐scale macrovessels that we termed “mother vessels”. In contrast to most previous approaches that were restricted to microvascular networks or simple endothelialized channels [32], the printed vessel constructs described here closely resembled macrovessels, approaching the realistic dimensions of native human vessels in the centimeter range, and spontaneously developed key structural hallmarks of native vessel walls within just a few days after printing.

Within just one week, hiMPCs underwent differentiation into vascular cell types and self‐organized into early endothelial and mural layers reminiscent of embryonic vascular development. The constructs exhibited a characteristic tri‐layered organization consisting of a luminal CD31^+^ endothelial lining, an intermediate αSMA^+^ medial layer and a loosely organized adventitia‐like outer layer which also harbored CD150^+^/CD45^+^/CD34^+^ progenitor cells. This vessel wall‐like structure was surrounded by a thick vascularized compartment also containing CD150^+^/CD45^+^/CD34^+^ progenitor cells that are considered to differentiate into both hematopoietic and endothelial cells. This pattern is similar to early embryonic vessel formation [33]. Strikingly, IBA1^+^ macrophage‐like cells appeared despite their absence from the initial cell suspension. This finding indicates that the vascular constructs enable intrinsic differentiation of hiMPCs not only into vascular lineages but also non‐vascular cell types, an aspect that will be investigated in detail in future studies. This is of particular importance since macrophages orchestrate angiogenesis and vascular remodeling, e.g. by release of proangiogenic mediators such as VEGF. Furthermore, macrophages are critical for tissue homeostasis as well as plasticity. This intrinsic driven spontaneous differentiation of different cell types from a single progenitor source distinguishes this system from most engineered vascular models today, which mainly rely on differentiation into different cell types prior bioprinting [34, 35].

To mimic prevascularized tissue modules and advance the concept of modular vascularization, prevascularized mesodermal organoids were co‐cultured with the bioprinted “mother vessels”. The organoids contained intrinsic microvascular networks and gradually fused with the mesodermal tissue surrounding the outer “mother vessel” wall forming a continuous, vascularized tissue envelope. Microvessels extending from the organoids toward the bioprinted wall indicated early structural but not yet functional interconnection. This demonstrates that prevascularized organoids may serve as biological building blocks. In combination with bioprinted large “mother vessels” as perfusable conduits, this may promote formation of hierarchically organized vascular networks capable of supplying larger engineered tissue constructs. Controlled perfusion may not only promote further functional vascular integration between the “mother vessel” and the surrounding fused organoids but also counteract lumen narrowing of the printed “mother vessels”. Furthermore, these biomechanical stimuli are expected to enhance vascular maturation, resembling the effects induced by the onset of the heartbeat during embryonic development [36].

**FIGURE 8:**
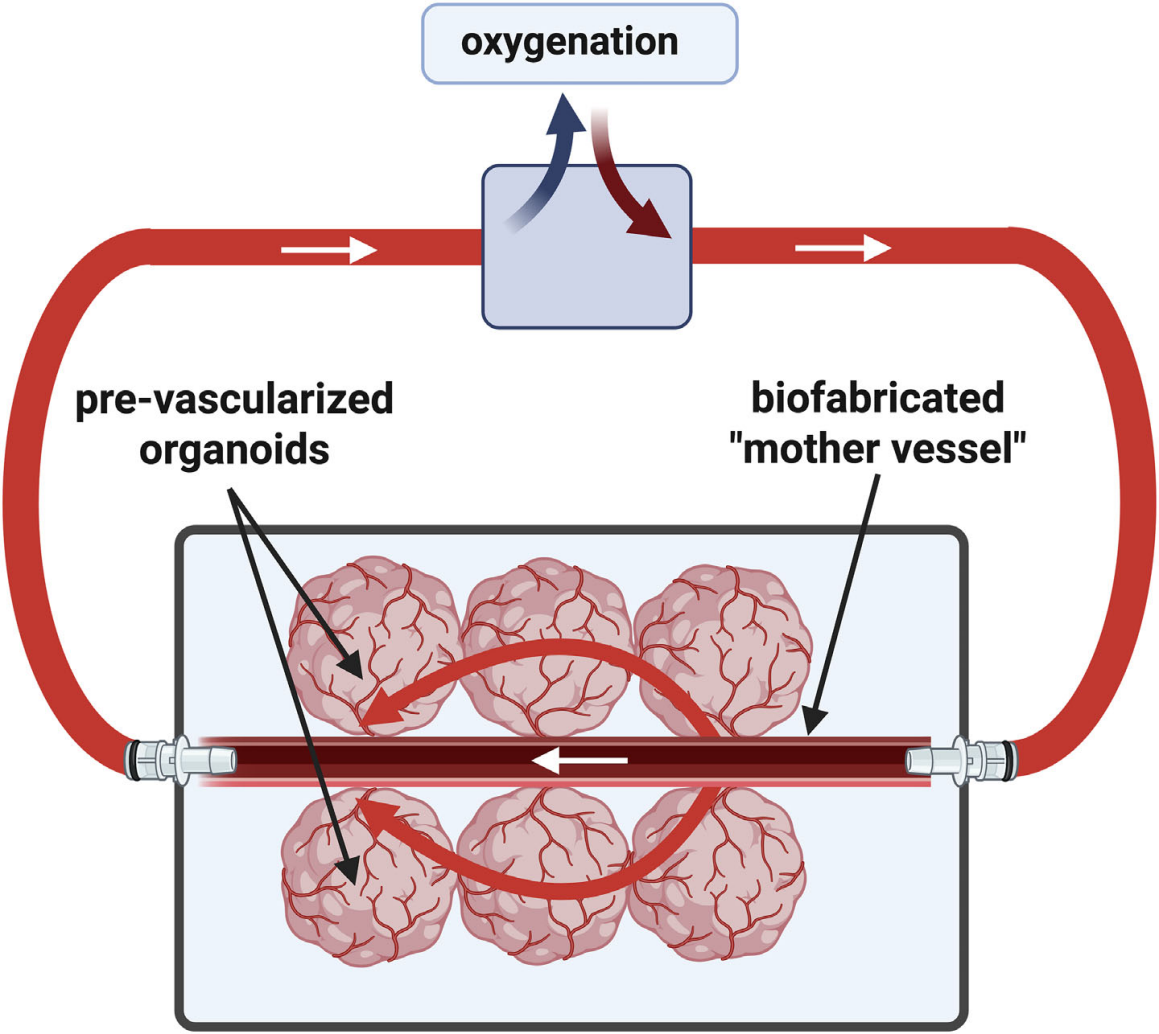
This graphical representation illustrates how the pre‐vascularized organoids are connected to the mother vessel via their own intrinsic vascular network, as demonstrated in this manuscript. In addition, it provides a future perspective on how such tissue constructs could be integrated into a perfusion system (as shown using a perfusion chamber in the proof‐of‐concept video in Video S3). This approach enables the establishment of large‐scale vascularized tissue models that are suitable for experimental perfusion through the mother vessel at the macrovascular level. Created in BioRender. Wörsdörfer, P. (2026) https://biorender.com/9rvca4s.

Taken together, this study presents a bioprinting strategy that enables the generation of centimeter‐scale, self‐organizing vascular tubes directly from hiMPCs in a single bioprinting step. The optimized bioink supports high viability, spatial organization, and intrinsic cell differentiation into vascular but also non‐vascular cells, reflecting key processes of embryonic vessel development. Integrating these printed “mother vessels” with prevascularized organoids establishes a modular system for hierarchical vascularization of large tissue constructs. In this system, macrovascular conduits enable vascular integration with the host or a perfusion system, while microvascular networks self‐assemble within the tissue to provide nutrient and oxygen supply, thereby addressing one of the major obstacles in tissue biofabrication. Using the newly designed perfusion chamber system, the mother vessel could be successfully perfused under pulsatile basal medium flow in a proof‐of‐concept experiment without detectable leakage. Although these experiments were limited in duration, they demonstrate the mechanical and structural stability and perfusability of the bioprinted construct. Future experiments using prolonged, optimized perfusion with pulsatile volume loading and shear stress are expected to further promote and complete vessel wall maturation by introducing these physiologically relevant mechanical cues. Furthermore, such stimuli will improve the tightness of the endothelial barrier as well as endothelial‐smooth muscle interactions, which serve as the basis for more sophisticated long‐term functional studies, such as endothelial‐dependent vasodilation and endothelial barrier leakiness. Together, our present data support the suitability of the mother vessel construct for establishing an in vitro circulatory system capable of supplying large‐scale, complex tissue constructs (Figure 8). Moreover, this platform would enable tissue‐ and organ‐specific analyses at molecular, cellular, and humoral levels.

## Conflicts of Interest

The authors declare no conflicts of interest.

## Supporting information




**Supporting File 1**: advs76018‐sup‐0001‐SuppMat.docx.

**Supporting File 2**: advs76018‐sup‐0002‐VideoS3.mp4.

**Supporting File 3**: advs76018‐sup‐0003‐VideoS4A.mp4.

**Supporting File 4**: advs76018‐sup‐0004‐VideoS4B.mp4.

**Supporting File 5**: advs76018‐sup‐0005‐VideoS4C.mp4.

## Data Availability

This study does not include any original code. Information necessary to reproduce or reanalyze the data presented in this work is available from the corresponding author upon reasonable request.
