# Supplementary material for: Generation of Self‐Organizing Macrovascular Constructs by Bioprinting Human iPSC‐Derived Mesodermal Progenitor Cells
Source: Adv Sci (Weinh). 2026 Jun 9;13(49):e76018. doi: 10.1002/advs.76018 (PMC13336730; doi:10.1002/advs.76018)
Supplement: Supplementary file 1 [file ADVS-13-e76018-s001.docx]

**Supplementary Information**

**Figure S1: Rheological characterization of the XG-based embedding medium. (A)** Shear stress recovery over time. **(B)** Flow curve of viscosity as a function of shear rate. **(C)** Frequency sweep test showing the gel-like and viscoelastic liquid-like behavior of the support bath.

**Figure S2: Microstructural changes in FGXC overtime.**

FGXC samples without cells were incubated in PBS for 6 days. Changes in the polymeric network density were observed, suggesting swelling of the hydrogel and release of XG over time.

**Figure S3**: This video visualizes the three-dimensional architecture and structural organization of the whole printed construct, including the properly formed vessel wall and vascular sprouts within the whole construct after immunostaining for CD31 and SMA and subsequently tissue clearing. The printed mother vessel construct is opened on a side for being able to image and to make video recording from the luminal surface of the vessel wall.

**Figure S4A:** This video demonstrates perfusion of the mother vessel tube, which is clamped at both ends (indicated by doted orange lines) to the inlet and outlet openings of the metal tubing. Perfusion was achieved using a peristaltic pump, with basal culture medium flowing through the construct containing the mother vessel and exiting at the open end of the tubing as visible in the video.

**Figure S4B:** This video recording in higher magnification demonstrates the pulsatile perfusion of the mother vessel tube within the section marked by doted lines.

**Figure S4C:** This high-magnification video recording demonstrates the absence of detectable leakage through the mother vessel wall.
